# Facile synthesis of a covalent organic framework for efficient solid-phase extraction and analysis of carbamate pesticide residues in fruits

**DOI:** 10.1016/j.fochx.2026.104666

**Published:** 2026-10-24

**Authors:** Rana Morsi, Shaikha S. AlNeyadi, Bayan Al-Meetani, Osama Shekhah, Mohammed A. Meetani

**Affiliations:** a Chemistry Department, College of Science, United Arab Emirates University, Al-Ain, P.O. Box 15551, United Arab Emirates; b Chemical Engineering Department, College of Engineering, United Arab Emirates University, Al-Ain, P.O. Box 15551, United Arab Emirates; c Advanced Membranes and Porous Materials Center Physical Science and Engineering Division, King Abdullah University of Science and Technology, Thuwal 23955-6900, Saudi Arabia

**Keywords:** Solid-phase extraction, Carbamates, Covalent organic frameworks, UHPLC-MS/MS, Fruits

## Abstract

A triazine-based covalent organic framework (COF-RC) was synthesized via Schiff-base condensation between 2,4,6-trihydroxybenzene-1,3,5-tricarbaldehyde and 1,3,5-tris(4-aminophenyl)triazine and applied as a sorbent for the solid-phase extraction of seven carbamate pesticides from fruits. COF-RC was characterized by PXRD, FTIR, nitrogen sorption, and TGA, confirming a crystalline microporous framework with a BET surface area of 910 m^2^/g, a pore size of 16.0 Å, and high chemical and thermal stability. Key SPE parameters, including elution conditions, pH, ionic strength, sorbent amount, adsorption and desorption times, and shaking speed, were systematically optimized. The developed SPE-UHPLC-MS/MS method achieved recoveries of 92.7–102.9% across dates, tomatoes, grapes, and apples, with excellent linearity (R^2^ = 0.9985–0.9997), LODs of 0.010–0.051 ng/g, and LOQs of 0.031–0.155 ng/g. These results demonstrate the suitability of COF-RC as an efficient sorbent for trace-level carbamate determination in food and highlight its potential for food safety monitoring.

## Highlights

• A novel COF-RC was synthesized for efficient carbamate pesticide extraction.
• COF-RC showed high crystallinity, microporosity, and chemical stability.
• The COF-RC-SPE method achieved excellent recoveries (92.7–102.9%).
• Ultra-low LODs (0.01–0.051 ng/g) were obtained using HPLC-MS/MS analysis.
• COF-RC enabled selective and sensitive carbamate detection in fruit samples.

## 1 Introduction

Carbamate pesticides have gained widespread agricultural use due to their broad biological activity against various pests, relatively short environmental persistence, and lower toxicity compared with some other classes of pesticides (Chowdhury et al., 2014). Their mode of action involves inhibition of the acetylcholinesterase (AChE) enzyme, leading to disruption of nerve impulse transmission and eventually causing pest mortality. Despite being effective in crop protection, their residues pose threats to human health and non-targeted species through environmental exposure and food chain contamination, particularly in fruits, vegetables, and dairy products. As AChE inhibitors, carbamates cause neurotoxicity and can disrupt immune and metabolic functions, posing chronic health risks (Bini Dhouib et al., 2016; Fatunsin et al., 2020; Morsi et al., 2024). Therefore, environmental agencies and regulatory authorities worldwide have established Maximum Residue Limits (MRLs) to control pesticide residues in food.

Various analytical techniques have been employed for the determination of carbamate residues in food, including fluorescence-based methods, liquid chromatography (LC), and gas chromatography (GC) coupled with different detectors (Chen et al., 2020; Guo et al., 2022; Morsi et al., 2024; Sarawan & Chanthai, 2014; Wang et al., 2019). Among these, LC coupled with tandem mass spectrometry (LC-MS/MS) is widely employed because of its high sensitivity, selectivity, and quantitative capability (Masiá et al., 2016). However, the complex nature of food matrices combined with the trace-level concentrations of carbamates makes direct analysis challenging. Consequently, efficient sample pretreatment, including clean-up, extraction, and concentration steps, is crucial for the accurate determination of carbamate residues in various food samples (Tang et al., 2016). However, the effectiveness of these procedures largely relies on the choice of sorbent material, which directly influences recovery, selectivity, and reproducibility.

Among the available sample preparation approaches, solid-phase extraction (SPE) is widely used because of its simplicity, adaptability, reduced organic-solvent consumption, and high extraction efficiency (Askar et al., 2026; Badawy et al., 2022; Xu et al., 2021). Conventional SPE adsorbents such as C18, silica, activated carbon, and polymeric materials often lack selectivity, have low stability, and exhibit limited adsorption capacity. To overcome these limitations, various novel materials have been developed as SPE sorbents, including metal-organic frameworks (MOFs), carbon-based nanomaterials, and covalent organic frameworks (COFs), with the aim of achieving greater selectivity, improved adsorption capacity, and higher thermal, chemical, or mechanical stability (Andrade-Eiroa et al., 2016; Augusto et al., 2010). Among these emerging materials, COFs have attracted significant attention due to their unique structural properties, versatile functionalization capabilities, and exceptional stability.

COFs are crystalline porous materials constructed through covalent linkage of organic building units into two- or three-dimensional networks. Their high surface area, tunable pore structure, structural stability, and readily tailorable functionality make them particularly attractive as sorbents for sample preparation and extraction (Diercks & Yaghi, 2017; Elgharpawy et al., 2026; Qian et al., 2023; Usman et al., 2026). Despite the large number of reported applications of COFs, few have been specifically developed for sample extraction and clean-up in analytical applications (Torabi et al., 2022). Guo et al. (2022) demonstrated the effectiveness of COF-TpDB as an adsorbent for the SPE of four carbamate residues in apples and tomatoes via hydrogen bonding and π–π stacking, achieving recoveries ranging from 80.4 to 101.2%. Similarly, Xu et al. (2021) reported the excellent adsorption capability of TAPT-DHTA-COF toward phenoxy carboxylic acids (PCAs) in plant-derived foods through hydrogen bonding, halogen bonding, and π–π interactions, with recoveries from 81.2 to 107%. Furthermore, Gao et al. (2021) demonstrated that 3DGA@COF exhibited good adsorption capacity toward organophosphorus pesticides (OPPs), with recoveries between 75.40 and 102.13%. Despite these advances, relatively few COF-based sorbents have been reported for carbamate residue analysis, and reported approaches have generally been limited to a small number of target carbamates or a restricted range of matrices.

Carbamates are derived from carbamic acid, their structures may be aliphatic or incorporate aromatic moieties containing phenyl rings or heterocyclic systems. They are semi-polar compounds that can participate in hydrogen bonding, either by accepting a hydrogen bond via their carbonyl group or donating one via the amino group (Matošević & Bosak, 2020). Given these structural characteristics, a COF containing aromatic building blocks and hydrogen-bonding sites could serve as a suitable SPE sorbent. This may facilitate hydrogen bonding and π–π interactions between the COF's π-conjugated systems and phenyl rings in carbamates, thereby supporting carbamate retention during extraction.

This work aims to develop and validate a sensitive solid-phase extraction method employing a multifunctional triazine-based COF, denoted COF-RC, as a selective sorbent for the simultaneous determination of seven carbamate pesticides across four complex fruit matrices. This approach broadens the multiresidue scope and matrix applicability of previously reported COF-based carbamate extraction methods. COF-RC was synthesized via the condensation of 2,4,6-trihydroxybenzene-1,3,5-tricarbaldehyde (L1) and 1,3,5-tris(4-aminophenyl)triazine (L2), resulting in a 2D framework rich in nitrogen-containing groups, hydroxyl groups, and extended π-conjugated systems. The prepared COF-RC was characterized by powder X-ray diffraction (PXRD), Fourier-transform infrared spectroscopy (FTIR), nitrogen sorption measurements for surface area and porosity characterization, and thermogravimetric analysis (TGA). The SPE conditions were systematically optimized for the simultaneous extraction of carbosulfan, propoxur, pirimicarb, bendiocarb, fenoxycarb, carbofuran, and methiocarb, followed by ultra-high-performance liquid chromatography-tandem mass spectrometry (UHPLC-MS/MS) determination in date palm fruit, tomatoes, apples, and grapes. The analytical performance of the developed method was subsequently evaluated in terms of sensitivity, accuracy, precision, selectivity, matrix applicability, and sorbent reusability.

## 2 Materials and methods

### 2.1 Chemicals and reagents

High purity carbamate standards (carbosulfan, propoxur, pirimicarb, bendiocarb, fenoxycarb, carbofuran, and methiocarb) were obtained from Dr. Ehrenstorfer (Augsburg, Germany). Pirimicarb d6, ammonium formate, anhydrous magnesium sulfate (MgSO_4_), graphitized carbon black (GCB), acetic acid, formic acid (FA), 1,4-dioxane, o-dichlorobenzene, dimethylformamide (DMF), and acetone were purchased from Sigma-Aldrich (St. Louis, MO, USA). LC-MS grade acetonitrile and methanol were supplied by Honeywell (Seelze, Germany). Milli-Q Plus system (MilliporeSigma, USA) was used to produce ultrapure water. Millex polytetrafluoroethylene (PTFE) syringe filters were obtained from Merck Millipore (Carrigtwohill, Ireland). Ultrahigh-purity nitrogen (99.999%) supplied by Air Products was employed for the sorption experiments.

### 2.2 Synthesis of raw materials

#### 2.2.1 Synthesis of L1

The synthesis of L1 was performed as follows (Fig. S1): 1,3,5-trihydroxybenzene (3 g, 23.8 mmol) and hexamethylenetetramine (8.34 g, 59.5 mmol) were added to a round-bottom flask under a nitrogen atmosphere. The mixture was then heated to 100 °C and stirred for 2.5 h in trifluoroacetic acid (47 mL). Subsequently, hydrochloric acid (50 mL) was added, and the mixture was maintained at 100 °C for an additional 1 h. After cooling to room temperature, the filtrate was extracted with dichloromethane (3 × 100 mL), and the organic layer was separated and dried over anhydrous sodium sulfate. The crude product was obtained by rotary evaporation. The final product was collected after recrystallization from hot ethanol, yielding a pure white solid (620 mg, 18%); ^1^H NMR (DMSO‑*d*_6_, 400 MHz) (*δ, ppm*): 6.47 (br s, 3H, OH), 9.96 (s, 3H, CHO) (Fig. S2); ^13^C NMR (DMSO‑*d*_6_, 100 MHz) (*δ, ppm*): 103.7, 174.1, 191.6 (Fig. S3).

#### 2.2.2 Synthesis of L2

The synthesis of L2 was performed with slight modifications to the procedure reported by Fajal et al. (2023) (Fig. S4). In a 50 mL round-bottom flask placed in an ice bath, 4-aminobenzonitrile (2 g) was treated with triflic acid (2 mL), which was added dropwise. The mixture was stirred at room temperature for 48 h, after which it was poured into 200 mL ice-cold water and neutralized with 2 M sodium hydroxide solution. During neutralization, the solution became clear and deep yellow, and a deep yellow precipitate formed immediately upon reaching neutrality. The precipitate was collected by filtration, thoroughly washed with water, and dried under vacuum to constant weight, yielding a pure yellow solid (1.6 g, 91%); ^1^H NMR (DMSO‑*d*_6_, 400 MHz) (*δ, ppm*): 8.96 (d, *J* = 8.8 Hz, 6H), 8.17 (d, *J* = 8.4 Hz, 6H), 5.28 (s, 6H) (Fig. S5); ^13^C NMR (DMSO‑*d*_6_, 100 MHz) (*δ, ppm*): 147.1, 131.8, 130.9, 116.5, 114.9 (Fig. S6).

### 2.3 COF-RC synthesis

The synthesis of COF-RC was carried out by combining L1 (1 mmol), L2 (1.5 mmol), o-dichlorobenzene (0.9 mL), and butanol (0.1 mL) in a tube, followed by sonication for 2 min. Subsequently, 0.3 mL of 6 M acetic acid was added to the mixture. The reaction mixture was then transferred to an autoclave and heated at 120 °C for 5 days in a static oven. After the reaction, the mixture was allowed to cool to room temperature and washed sequentially with DMF (30 mL) and chloroform (30 mL). The resulting product was filtered and dried under vacuum at 60 °C, yielding the COF as a fluffy powder.

### 2.4 Instrumental analysis

Thin-layer chromatography (TLC) was performed on silica gel glass plates (Silica gel, 60 F254, Fluka, Merck, Darmstadt, Germany), and column chromatography was conducted using Kieselgel S (silica gel S, 0.063–0.1 mm, Merck, Darmstadt, Germany). Melting points were determined using a Gallenkamp apparatus (Toledo, OH, USA) and were corrected. Elemental analysis was carried out with a Leco Model CHN-600 elemental analyzer (Ontario, Canada). FTIR spectra were obtained using KBr pellets on a Thermo Nicolet model 470 FTIR spectrophotometer (Thermo Scientific, Waltham, MA, USA). Nuclear magnetic resonance (NMR) spectra were recorded on a Varian 400 MHz spectrometer (^1^H NMR at 400 MHz and ^13^C NMR at 100 MHz; Agilent Technologies, Santa Clara, CA, USA) with dimethyl sulfoxide‑*d*_6_ (DMSO‑*d*_6_) as the solvent. Tetramethylsilane (TMS) served as an internal reference, with chemical shifts reported in parts per million (*δ*, ppm). PXRD analysis was conducted using a Shimadzu-6100 diffractometer (Shimadzu-series, Kyoto, Japan) with Cu-Kα radiation (λ = 1.542 Å). Diffraction data were collected over a 2θ range of 20–50° at a rate of 1°/min, under room temperature and atmospheric pressure. Nitrogen sorption measurements and pore-size analyses were performed using a PMI BET Sorptometer (BET-201-AEL, PMI, USA) at 77 K using a liquid nitrogen bath. TGA was performed on a 0.2 g sample, heated to 600 °C at a rate of 5 °C/min to monitor weight changes as a function of temperature.

The chromatographic separation of carbamate pesticides was performed using a UHPLC instrument (Nexera-i LC-2040C 3D, Kyoto, Japan) with an ACQUITY UPLC BEH C18 column (2.1 mm × 150 mm, 1.7 μm) supplied by Waters (Milford, USA) at 44 °C. The mobile phase was water with 10 mM ammonium formate (pH 3) (A) and a mixture of methanol and acetonitrile (2:1, v/v) (B) and the flow rate was set at 0.18 mL/min. Gradient elution was performed as follows: 0–4.0 min, 12% B; 4.0–5.50 min, 12–88% B; 5.50–13 min, 88% B; 13.0–13.50 min, 88–96% B; and 13.50–15.50 min, 96% B; 15.50–17.0 min, 96–12% B; 17.0–20.0 min, 12% B. MS analysis was performed on a Shimadzu 8030 triple quadrupole mass spectrometer (Kyoto, Japan) with an electrospray ionization (ESI) source in multiple reaction monitoring (MRM) mode. Detailed MS/MS parameters are provided in Table S1.

### 2.5 SPE procedure

The SPE procedure was performed by adding 10 ± 0.01 mg of COF-RC to the sample solution. The mixture was first sonicated (Branson 5800, Branson Ultrasonics, USA) for 5 min at 25 °C to ensure proper COF dispersion, followed by shaking at 400 rpm (VWR OS-500 Orbital Shaker, VWR International, USA) for 30 min at 25 °C. After centrifugation at 4000 rpm for 10 min, the supernatant was discarded. Analyte desorption was achieved using 2.5 mL methanol under 15 min of sonication. The mixture was centrifuged under the same conditions, and the supernatant was collected and evaporated. The extract was reconstituted with 1 mL of the organic mobile phase and filtered through a 0.45 μm PTFE syringe filter prior to UHPLC-MS/MS analysis. All analyses were performed in triplicate.

### 2.6 Optimization of SPE conditions

To maximize the extraction efficiency of the seven carbamate pesticides, various parameters affecting the SPE performance were optimized: type of elution solvent, solvent volume, sample pH, ionic strength, COF amount, adsorption time, desorption time, and shaking rate. For each optimization experiment, a 50 ng/g carbamate standard solution was used to evaluate extraction efficiency based on the response ratio of each carbamate pesticide to the internal standard. All experiments were performed in triplicates at room temperature.

### 2.7 Selectivity evaluation

The extraction selectivity of COF-RC toward carbamate pesticides was evaluated by conducting competitive extraction experiments using a mixture of target carbamates (carbosulfan, propoxur, pirimicarb, bendiocarb, fenoxycarb, carbofuran, and methiocarb) and OPPs (chlorpyrifos, diazinon, malathion, parathion, phorate, dicrothophos, triazophos, and temephos). The OPPs were selected as potential interferents because of their common co-occurrence with carbamate pesticides in fruit sample. They represent a range of structural classes, polarities, and molecular features, enabling an assessment of COF-RC selectivity under competitive extraction conditions. The experiments were conducted under optimized SPE conditions at pH 6, with each compound present at 50 ng/g in the test mixture. The selectivity of the sorbent was evaluated by comparing the recoveries of carbamates and OPPs under identical extraction conditions.

### 2.8 Sample preparation

Fresh dates, tomatoes, grapes, and apples were purchased from local markets in Al Ain, UAE. The samples were enclosed in sterile polypropylene bags to prevent contamination during transport to the laboratory. Upon arrival, the samples were stored at 4 °C to maintain their freshness until analysis. Sample handling followed the European SANTE/11312/2021 guidelines.

Each sample was cut into small pieces and homogenized using a food blender. For dates and apples, deionized water (1:1, *v*/v) was added during homogenization to obtain a suitable consistency. A 5.00 ± 0.01 g of the homogenized sample was weighed into a 50 mL centrifuge tube, and 5 mL of acetonitrile was added. The mixture was vortexed for 1 min and sonicated for 5 min. Then, 3.00 ± 0.01 g of anhydrous MgSO₄ was added (1.50 g for dates), and the mixture was vortexed and centrifuged at 6000 rpm for 5 min. The supernatant was transferred to a new 50 mL centrifuge tube, and the extraction was repeated twice without the addition of further MgSO_4_. The combined supernatants were treated with 400 ± 0.01 mg of GCB for clean-up and centrifuged at 6000 rpm for 5 min. The resulting supernatant was collected into a clean glass centrifuge tube and evaporated to dryness. The dried extract was reconstituted with 10 mL of deionized water (pH 6), and the COF-RC-based SPE procedure was then performed.

## 3 Results and discussion

### 3.1 Synthesis and structural characterization of COF-RC

The synthesis of COF-RC was achieved through a Schiff-base condensation reaction between L1 and L2 in an o-dichlorobenzene/butanol (9:1, v/v) solvent mixture, using 6 M acetic acid as a catalyst at 120 °C for 5 days. Fig. 1 shows the synthetic route for COF-RC formation via condensation between the aldehyde and amine groups of the precursors, resulting in a 2D framework connected via imine linkages. The formation of COF-RC was confirmed by multiple characterization techniques, including PXRD, FTIR, nitrogen adsorption–desorption isotherms, and TGA. COF-RC was synthesized in multiple independent batches, and consistent PXRD patterns were obtained across batches, demonstrating good batch-to-batch structural reproducibility.

**Fig. 1:**
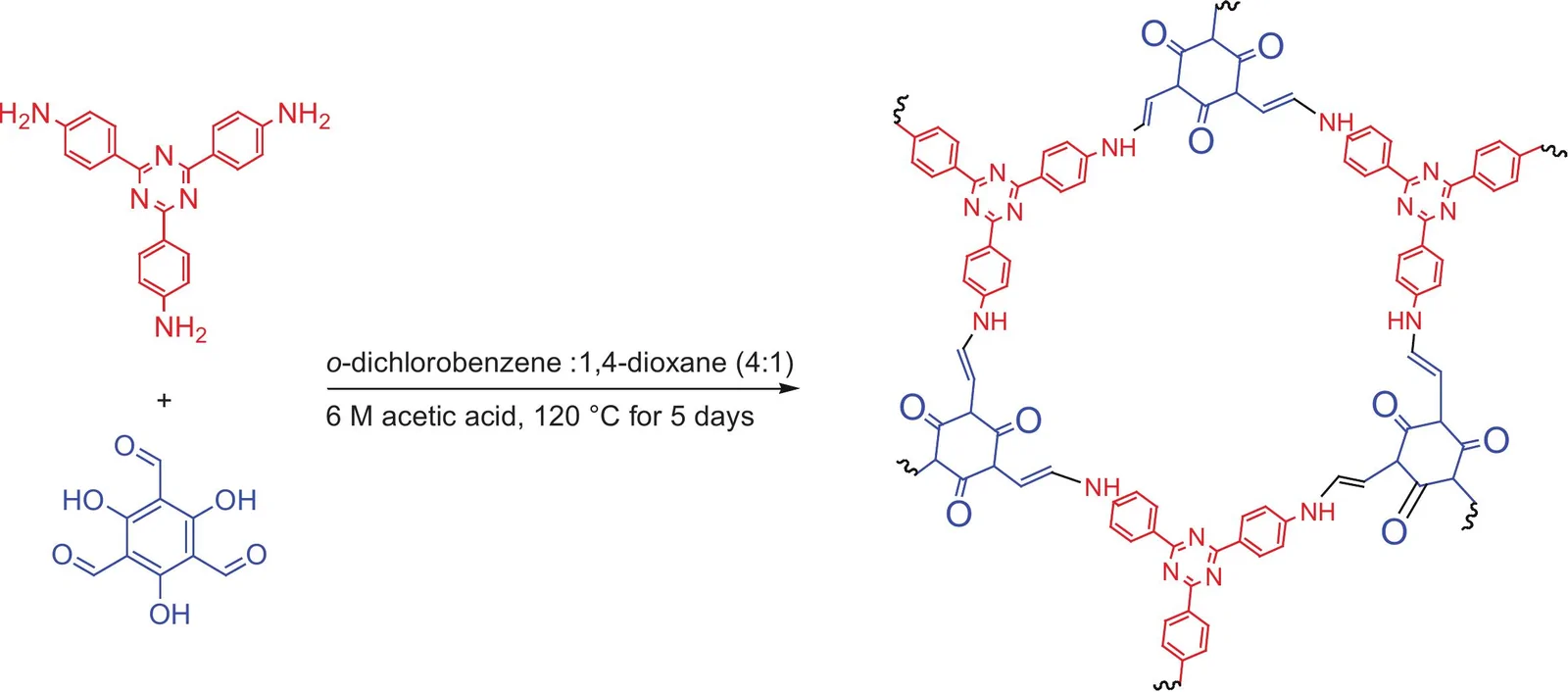
Illustration of the solvothermal synthesis of COF-RC via Schiff-base condensation between the aldehyde groups of L1 and the amino groups of L2. The initially formed imine intermediates undergo enol–keto tautomerization, yielding a chemically stable two-dimensional β-ketoenamine-linked covalent organic framework.

#### 3.1.1 Porosity and surface area analysis

Nitrogen adsorption–desorption isotherms (Fig. 2A) exhibited a Type I isotherm, confirming the microporous nature of COF-RC, with a high BET surface area of 910 m^2^/g and a pore volume of 1.10 cm^3^/g. The pore size distribution (Fig. 2B) showed a sharp peak at 16.0 Å, indicating a relatively uniform pore-size distribution. These characteristics support the suitability of COF-RC as an SPE sorbent for carbamate pesticides, as its high surface area provides abundant adsorption sites, while its porous network may facilitate molecular diffusion and analyte retention.

**Fig. 2:**
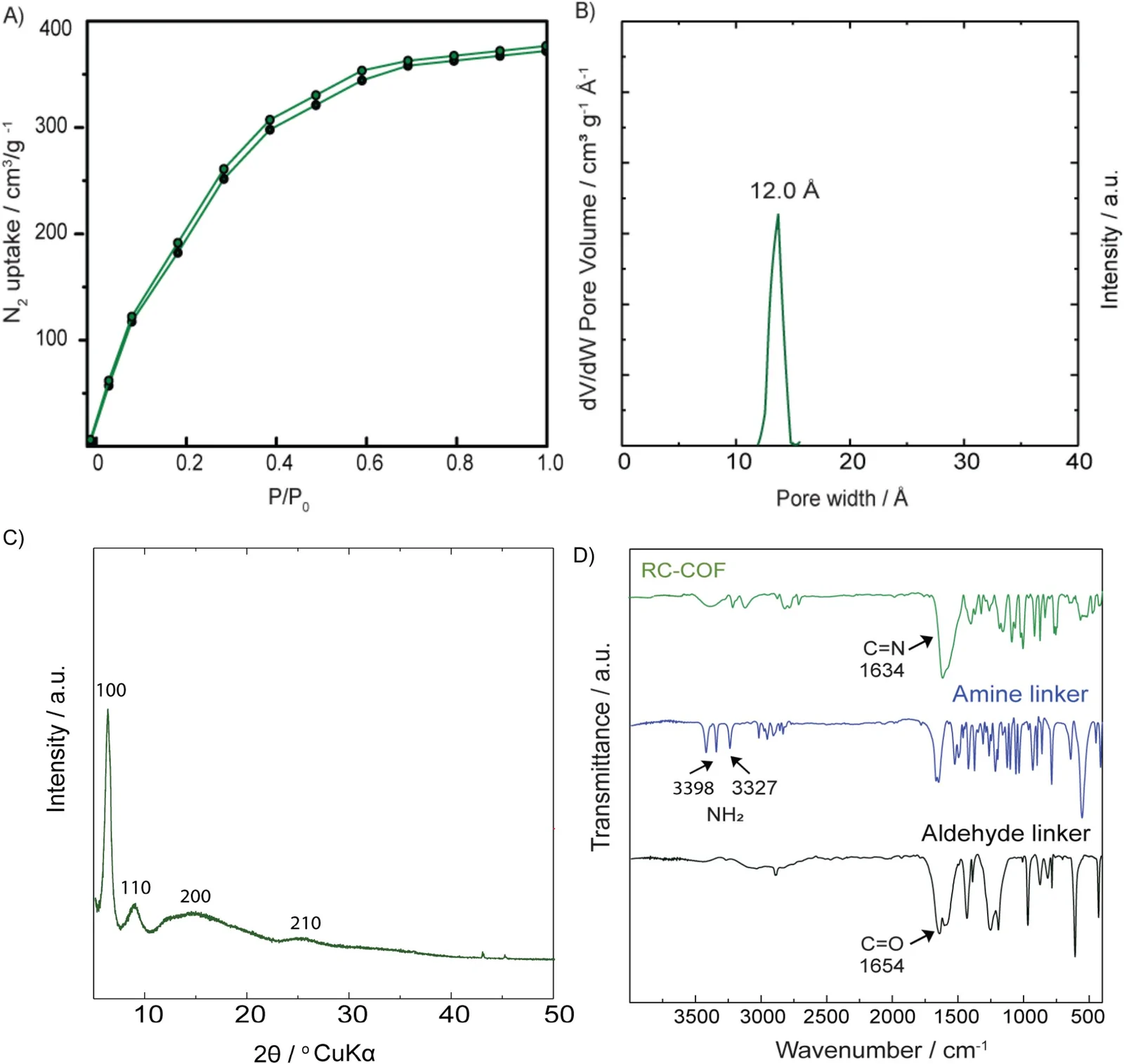
Characterization of COF-RC: (A) Type I nitrogen adsorption-desorption isotherm, indicating microporosity; (B) Pore size distribution with a dominant 16.0 Å pore width; (C) Experimental PXRD pattern of COF-RC showing distinct reflections at 2θ ≈ 6.9° (100), 11.9° (110), 20.3° (200), and 25.7° (210), characteristic of a 2D layered crystalline framework. The presence of these indexed peaks confirms the long-range order and periodic pore structure of COF-RC.; and (D) FTIR confirming imine (-C=N) linkages and functional group incorporation.

#### 3.1.2 PXRD data and structural modeling

The crystallinity and long-range order of COF-RC were verified by PXRD analysis (Fig. 2C). The experimental pattern showed a sharp and intense peak at 2θ ≈ 6.9°, indexed to the (1 0 0) reflection, together with weaker peaks assigned to the (1 1 0), (2 0 0), and (2 1 0) planes. These characteristic reflections confirm the successful formation of a highly ordered 2D layered framework, consistent with earlier reports of 2D COFs that exhibit similar low-angle reflections (Nagai et al., 2011; Pelkowski et al., 2023). To further elucidate the stacking arrangement, the experimental PXRD profile was compared with simulated patterns for AA (eclipsed) and AB (staggered) stacking models (Fig. S7). The AB-stacked model exhibited excellent agreement with the experimental data, particularly in reproducing the dominant (1 0 0) peak and the relative positions of higher-order reflections. In contrast, the AA-stacked simulation predicted sharper and more intense peaks not observed experimentally. This strongly suggests that COF-RC layers preferentially adopt an AB-staggered stacking mode, a configuration commonly observed in 2D COFs due to reduced steric repulsion and enhanced framework stability (Pütz et al., 2020).

The corresponding structural models and simulated PXRD patterns are shown in Fig. S8, in which both AA and AB stacking configurations were examined. The AB-stacking model not only reproduced the main low-angle diffraction peak but also matched well with additional reflections, confirming it as the most probable structural arrangement for COF-RC. The broadening of the experimental peaks relative to simulation is attributed to nanoscale crystallite size and partial interlayer disorder, both typical of solvothermal COF synthesis. Nevertheless, the clear appearance of multiple indexed peaks supports the presence of long-range periodicity and good crystallinity in COF-RC, consistent with the proposed structural model.

#### 3.1.3 Structural and thermal stability

The chemical stability of COF-RC was evaluated by exposing it to dimethyl sulfoxide (DMSO), *N*,*N*-dimethylformamide (DMF), ethanol, acidic (HCl, pH 1) and basic (NaOH, pH 13) solutions, and boiling water (100 °C). The PXRD patterns remained essentially unchanged after exposure (Fig. S9A), confirming the excellent structural stability and chemical robustness of COF-RC under harsh conditions.

TGA (Fig. S9B) showed that COF-RC remained thermally stable up to approximately 500 °C, with only slight weight loss below 100 °C, attributed to the removal of physically adsorbed solvent molecules and moisture. The major weight loss observed above 500 °C was attributed to the thermal decomposition of the β-ketoenamine-linked covalent organic framework and collapse of its extended polymeric network.

#### 3.1.4 FTIR spectroscopy

FTIR spectroscopy (Fig. 2D) confirmed the successful formation of COF-RC through the condensation of L1 and L2. The disappearance of the characteristic aldehyde (C=O) stretching band at 1654 cm^−1^ and the marked reduction of the primary amine (N—H) stretching vibrations at 3398 and 3327 cm^−1^ indicated the consumption of the monomer functional groups during polymerization. The characteristic absorption band at 1634 cm^−1^ is consistent with the conjugated β-ketoenamine framework formed after Schiff-base condensation and subsequent enol–keto tautomerization. The β-ketoenamine linkage, together with the triazine nitrogen atoms and hydroxyl functionalities within the framework, provides multiple interaction sites for carbamate pesticides through hydrogen bonding and π–π interactions with their aromatic moieties, thereby enhancing the selective retention of carbamates by COF-RC.

### 3.2 Adsorption mechanism

Carbamate pesticides contain carbonyl (C=O), ether (C–O–C), aromatic, and amino functional groups that strongly influence their interactions with porous sorbent materials. Understanding these interactions is essential for rationalizing the selective extraction performance of COF-RC. In this study, propoxur was selected as a representative carbamate to investigate its adsorption behavior on the synthesized COF-RC. Owing to its β-ketoenamine-linked framework, triazine nitrogen atoms, hydroxyl functionalities, and extended π-conjugated aromatic network, COF-RC provides multiple active sites capable of interacting with carbamate molecules through hydrogen bonding, π–π stacking, and electrostatic interactions. These synergistic interactions contribute to the high adsorption efficiency and selectivity observed for carbamate pesticides.

The adsorption of propoxur onto COF-RC is primarily governed by a combination of hydrogen bonding, π–π stacking interactions, and electrostatic attraction. The β-ketoenamine linkage together with the hydroxyl groups and electron-rich triazine nitrogen atoms provides multiple adsorption sites capable of interacting with the carbonyl, ether, and amino functionalities of propoxur. The hydroxyl groups of COF-RC act predominantly as hydrogen bond donors toward the carbonyl and ether oxygen atoms of propoxur, whereas the nitrogen atoms within the β-ketoenamine and triazine framework serve as hydrogen bond acceptors for the N—H group of the carbamate molecule. These complementary donor–acceptor interactions significantly enhance the adsorption strength and stabilize the adsorbed species.

Besides hydrogen bonding, strong π–π stacking interactions occur between the aromatic rings of propoxur and the highly conjugated aromatic framework of COF-RC. The overlap of the π-electron systems increases the binding affinity of the analyte within the porous framework and promotes selective retention of aromatic carbamate pesticides. In addition, electrostatic interactions arising from the electron-rich nitrogen-containing framework facilitate favorable orientation of propoxur molecules within the pores, further improving adsorption efficiency. The cooperative contribution of hydrogen bonding, π–π stacking, and electrostatic interactions is therefore responsible for the excellent extraction performance of COF-RC.

The adsorption process can be summarized as follows. Initially, propoxur molecules diffuse from the bulk solution toward the porous COF surface. The analyte is subsequently adsorbed through multiple hydrogen-bonding interactions involving the hydroxyl groups and nitrogen-containing sites of the β-ketoenamine framework, while simultaneous π–π stacking interactions occur between the aromatic rings of propoxur and the conjugated COF backbone. As adsorption proceeds, the available active sites gradually become occupied until adsorption equilibrium is established. The proposed adsorption mechanism is illustrated in Fig. S10.

To further investigate the adsorption mechanism, FTIR spectra of pristine COF-RC and propoxur-loaded COF-RC (COF-RC@Propoxur) were compared (Fig. S11). Noticeable shifts in the characteristic hydroxyl and framework vibration bands were observed after adsorption, indicating specific interactions between COF-RC and propoxur. The broad O—H stretching band shifted from 3458 cm^−1^ to 3490 cm^−1^, suggesting disruption of the original hydrogen-bonding network within the framework and the formation of new hydrogen bonds between the hydroxyl groups of COF-RC and the carbonyl and ether oxygen atoms of propoxur. In addition, the characteristic framework band near 1634 cm^−1^ shifted to 1644 cm^−1^ after adsorption, indicating changes in the local electronic environment of the conjugated β-ketoenamine framework resulting from interactions with propoxur. These spectral changes support the proposed adsorption mechanism involving hydrogen bonding and electrostatic interactions. The absence of new absorption bands after adsorption further indicates that the interaction between COF-RC and propoxur is dominated by non-covalent interactions rather than covalent bond formation, with hydrogen bonding and π–π stacking serving as the primary adsorption mechanisms.

To further investigate the interfacial interactions between COF-RC and propoxur, XPS survey spectra were obtained before and after adsorption (Fig. S12), while the principal binding energies determined from the corresponding high-resolution C 1s, N 1s, and O 1s spectra are summarized in Table S2. The principal C 1s and N 1s signals remained centered at approximately 284.5 and 399.6 eV, respectively, indicating no appreciable shifts in the dominant carbon and nitrogen environments. In contrast, the O 1s signal shifted from approximately 532.8 eV in pristine COF-RC to 532.4 eV after propoxur adsorption, corresponding to a shift of approximately 0.4 eV toward lower binding energy. This adsorption-induced shift indicates a modification of the local electronic environment of the oxygen-containing functionalities. Together with the observed FTIR spectral changes, the O 1s shift supports the involvement of oxygen-containing sites in the interfacial interactions between COF-RC and propoxur, consistent with the proposed hydrogen-bonding adsorption mechanism.

### 3.3 Optimization of SPE conditions

The key functional groups in COF-RC facilitate carbamates retention through π–π interactions and hydrogen bonding. To achieve high extraction efficiency, several key parameters affecting the SPE process were optimized: elution solvent type and volume, sample pH, ionic strength, COF amount, adsorption and desorption times, and shaking rate. Each parameter was investigated while keeping the other conditions constant, and extraction efficiency was evaluated based on the recoveries of the carbamate pesticides.

#### 3.3.1 Effect of elution solvent and volume

The elution efficiency of carbamate pesticides strongly depends on the type of elution solvent; thus, an appropriate elution solvent is required to efficiently desorb the carbamate pesticides from COF-RC. Five elution solvents were evaluated: methanol, methanol–water (3:1, v/v), acetonitrile, acetonitrile–water (3:1, v/v), and methanol–acetonitrile (1:1, v/v), with and without the addition of 0.1% FA. The addition of FA markedly decreased the recovery of carbosulfan to ≤20% in all solvent types (Fig. S13), while the other carbamates maintained acceptable recoveries (58–107%). In contrast, in the absence of FA, methanol provided maximum recoveries for all seven carbamates, including carbosulfan, with values approaching 100% (Fig. 3A). The observed difference in carbosulfan recovery indicates its sensitivity to acidic conditions; therefore, methanol without FA was selected as the elution solvent.

**Fig. 3:**
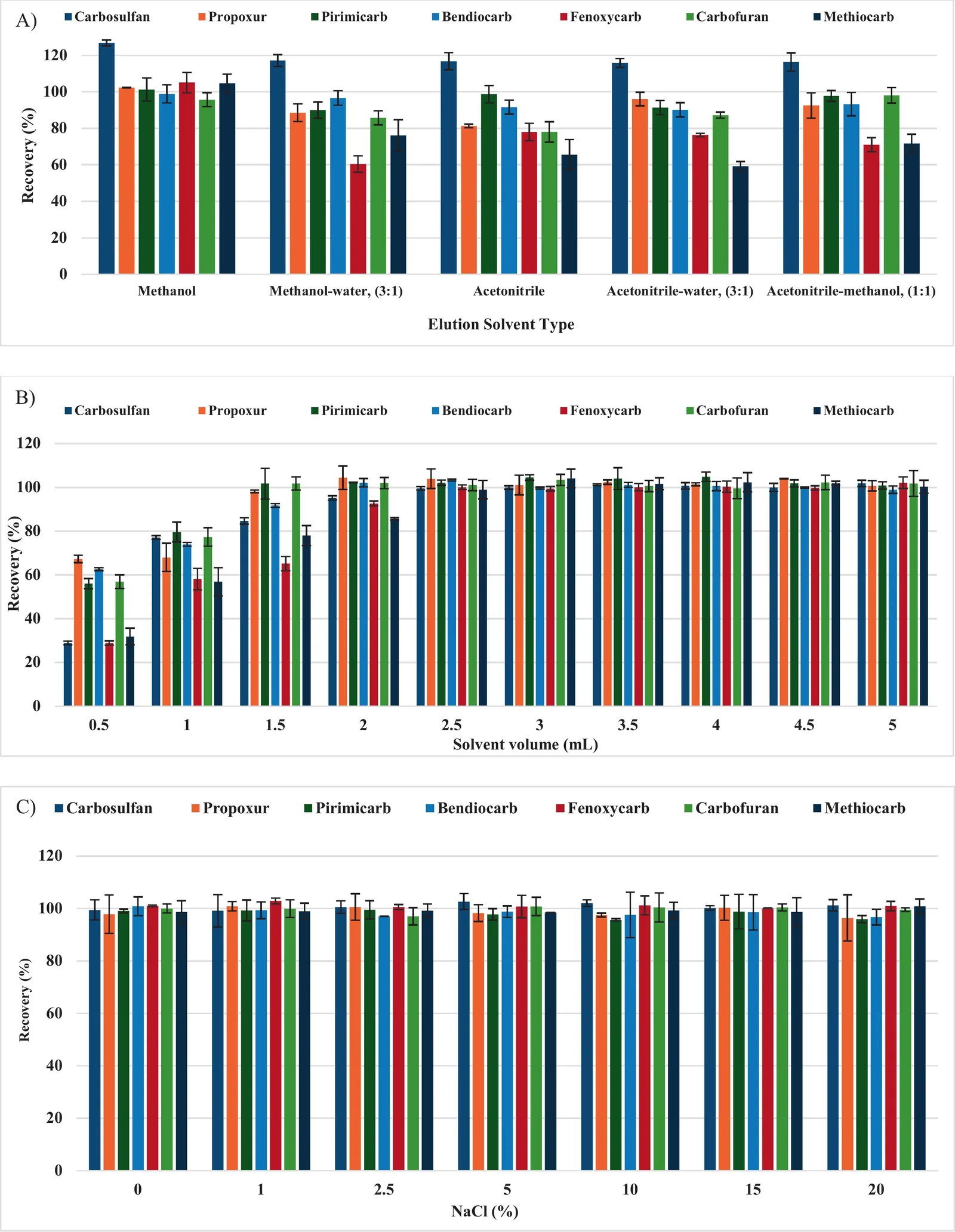
Effect of: (A) elution solvent type; (B) elution solvent volume; and (C) ionic strength for the SPE of carbamates using COF-RC as sorbent.

After selecting methanol as the optimal elution solvent, its volume was optimized to determine the minimum volume required for efficient desorption. Extraction recoveries were evaluated using methanol volumes of 0.5–5.0 mL. According to the results, 2.5 mL of methanol provided maximum extraction efficiency with recoveries ranging from 99 to 104% (Fig. 3B). As the methanol volume increased above 2.5 mL, recoveries remained constant with no significant improvement. Thus, 2.5 mL of methanol was selected as the optimal elution volume, resulting in high extraction efficiency while minimizing solvent consumption.

#### 3.3.2 Effect of ionic strength

The addition of salt can alter the ionic strength of the solution, affecting the solubility of analytes, solution viscosity, and mass transfer rate. High concentrations of salt typically decrease analyte solubility through salting-out effects, thus enhancing extraction efficiency. However, this can also lead to higher solution viscosity and reduced mass transfer rates. To investigate the impact of ionic strength on extraction efficiency, different concentrations of NaCl (0–20%) were evaluated. The results showed that the extraction efficiency was independent of salt addition, as all carbamates exhibited consistent recoveries (96–103%) across the studied salt concentration range (Fig. 3C). Therefore, subsequent SPE experiments were performed without the addition of salt.

#### 3.3.3 Effect of sample pH

The pH of the sample solution affects the extraction efficiency of carbamate pesticides by influencing both the interactions between the analytes and the sorbent and the molecular state of the analytes. Therefore, it is essential to determine the optimal pH to achieve high extraction recoveries while maintaining the stability of the carbamates. In this study, the effect of sample pH was investigated over the range of pH 4–10. As shown in Fig. 4A, most analytes showed low recoveries at pH 4, with a marked increase observed from pH 4 to pH 6. Maximum extraction efficiency was obtained at pH 6–7, with recoveries for all analytes approaching 100%. The recoveries gradually decreased for most analytes under basic conditions (pH 8–10), except for pirimicarb, which maintained stable recoveries over this range. Hence, pH 6–7 was selected as the optimal range for extracting carbamate pesticides.

**Fig. 4:**
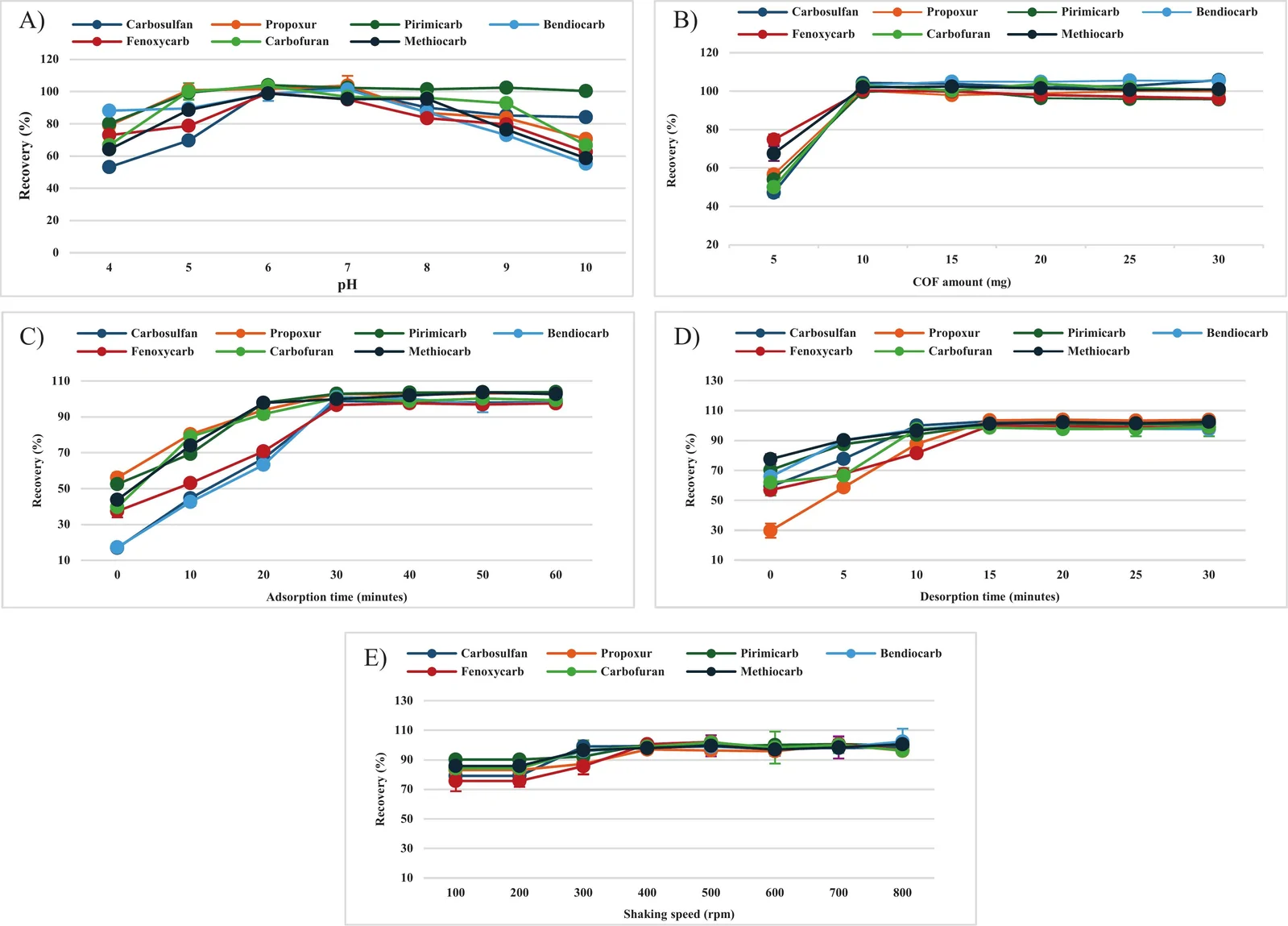
Effect of: (A) pH; (B) COF amount; (C) adsorption time; (D) desorption time; and (E) shaking speed on the SPE of carbamates using COF-RC as sorbent.

#### 3.3.4 Effect of COF amount

The number of available adsorption sites depends on the amount of sorbent, thereby affecting the extraction efficiency. Therefore, it is important to provide sufficient binding sites for the target carbamates while minimizing sorbent consumption. Different amounts of COF-RC (5–30 mg) were evaluated. The results showed low recoveries (47–75%) with 5 mg of sorbent, indicating an insufficient number of available interaction sites for effective analyte-COF interaction (Fig. 4B). A marked increase in recoveries for all compounds was observed with 10 mg of sorbent, with values ranging from 99 to 104%, indicating maximum extraction efficiency. Recoveries remained essentially unchanged as the COF-RC amount increased beyond 10 mg, indicating that 10 mg was the optimal sorbent amount for the extraction of carbamate pesticides.

#### 3.3.5 Effect of adsorption and desorption times

Adsorption time is another parameter that influences the overall extraction performance. It is important to determine the optimal time required for the sorbent to interact with the target analytes, while maximizing extraction efficiency and minimizing the total analysis time. The carbamate recoveries were evaluated at adsorption times of 0–60 min. A 5-min sonication step was applied to all samples to ensure uniform dispersion of the COF-RC in the solution prior to the adsorption time study. As shown in Fig. 4C, recoveries increased from 0 to 30 min and then remained essentially constant for all carbamates. At 30 min, the carbamates showed recoveries ranging from 97 to 103%, indicating that maximum extraction efficiency had been reached. Hence, 30 min was selected as the optimal adsorption time for carbamate pesticides.

It is also important to determine the optimal desorption time to ensure complete recovery of carbamate pesticides without unnecessarily prolonging the analysis time. The carbamate recoveries were investigated at desorption times of 0–30 min. Extraction recoveries increased from 0 to 15 min, at which maximum efficiency was reached with values approaching 100% for all carbamate pesticides (Fig. 4D). From 15 to 30 min, recoveries remained constant with no further improvement. Therefore, 15 min was selected as the optimal desorption time for efficient elution of the carbamate pesticides.

#### 3.3.6 Effect of shaking speed

Mass transfer between the analytes and the sorbent is affected by the shaking speed, influencing the extraction efficiency. Excessively high shaking speeds could potentially affect the COF structure, resulting in breakdown to smaller particles, framework damage, and potential loss of accessible interaction sites and surface area. On the other hand, low shaking speeds can result in insufficient contact between analytes and adsorbent, leading to reduced recoveries. Different shaking speeds ranging from 100 rpm to 800 rpm were studied to determine the optimal condition. As shown in Fig. 4E, 400 rpm provided maximum extraction efficiency with all carbamates showing recoveries in the range of 97–101%. Below 400 rpm, reduced recoveries were obtained due to insufficient mixing and mass transfer. Above 400 rpm, there was no significant improvement in recoveries. Hence, 400 rpm was selected as the optimal shaking speed.

### 3.4 Selectivity evaluation

Competitive SPE experiments were conducted to evaluate the selectivity of COF-RC. OPPs were selected not only because of their frequent co-occurrence with carbamates in agricultural products but also because of their structural features, which could potentially compete for COF-RC's binding sites. Some OPPs, such as chlorpyrifos and diazinon, contain aromatic rings that may interact with COF-RC's extended π-conjugated system via π-π stacking interactions, potentially competing with carbamates. Additionally, oxygen-containing functional groups in dicrotophos and malathion may participate in hydrogen bonding with the COF surface. Moreover, the phosphate groups in OPPs can act as HBA, interacting with the hydroxyl groups of COF-RC. However, these interactions are likely weaker compared to those with carbamates, which can act as both HBA (through the carbonyl group) and HBD (through the amino group), which may favor stronger interactions with COF-RC (Matošević & Bosak, 2020). The competitive SPE experiments demonstrated preferential extraction of carbamates over the tested OPPs. As shown in Table S3, all target carbamates were extracted efficiently, with recoveries ranging from 94.7% to 101.8%. In contrast, OPPs demonstrated significantly lower recoveries, ranging from 1.7% to 67.7%. Temephos was the only OPP with an acceptable recovery value (67.7%), which may be related to its larger aromatic structure and the potential for stronger π-π interactions with COF-RC. Additionally, compared to other polar OPPs, its relatively hydrophobic nature may also have contributed to its higher retention. Other OPPs exhibited recovery values below the acceptable range (60–140%) established by the European guidelines (European Commission, 2021).

The selectivity evaluation of COF-RC using propoxur as a representative carbamate together with the studied OPPs is presented in Fig. 5. The observed selectivity toward carbamates may be attributed to several factors, including the potential affinity between carbamates and COF-RC through hydrogen bonding, dipole-dipole interactions, and π-π stacking, as well as the relatively bulky structures of some OPPs, which may reduce their accessibility to the binding sites of COF-RC. The lower recoveries of the tested OPPs may therefore reflect differences in their ability to participate in hydrogen bonding and π-π interactions with COF-RC, together with possible steric effects. Overall, these results indicate preferential extraction of carbamates over the tested OPPs under the investigated conditions, supporting the suitability of COF-RC for selective carbamate extraction.

**Fig. 5:**
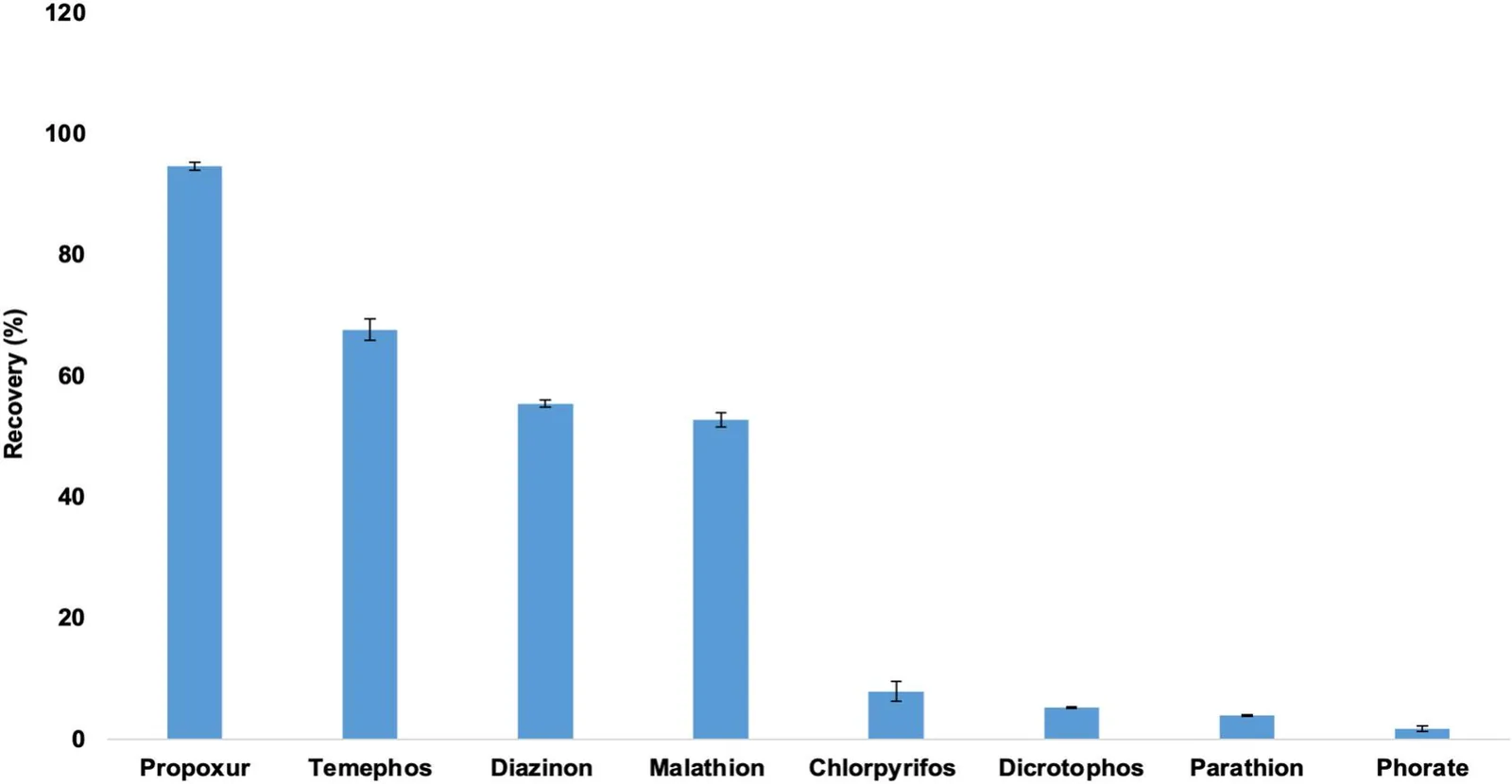
Selectivity evaluation of COF-RC.

### 3.5 Stability and reusability of COF-RC

The stability and reusability of COF-RC were evaluated over 15 consecutive extraction cycles using the same sorbent. Between each cycle, COF-RC was washed three times with 2.5 mL methanol, followed by 5 mL ultrapure water, and centrifuged to separate COF-RC from the washing solvent. As shown in Table S4, COF-RC maintained high extraction efficiency during the first 10 cycles, with recoveries ranging from 90 to 103%, indicating its robust performance under repeated use. After the 10th cycle, a gradual decrease in recoveries was observed for some carbamates, suggesting a partial loss in extraction efficiency. Among the studied carbamates, carbosulfan and fenoxycarb maintained relatively high recoveries after 15 cycles (93% and 81%, respectively), indicating stronger retention within the COF framework. Up to cycle 14, all carbamates maintained satisfactory recoveries (75–98%), indicating sustained extraction performance of COF-RC. However, by cycle 15, propoxur showed a significant decline in recovery (58%), while the other carbamates still maintained acceptable values (67–93%). These findings confirm that COF-RC can be reused for at least 10 cycles without a significant loss in its efficiency. This supports the durability and stability of COF-RC as an SPE sorbent. The observed loss in efficiency after the 10th cycle may be attributed to incomplete desorption of some residues during the washing steps, leading to partial blockage of interaction sites.

The structural stability of COF-RC after multiple reuse cycles was evaluated by PXRD and FTIR analyses. Notably, no significant changes were observed in the PXRD patterns or FTIR spectra before and after 10 reuse cycles. A slight reduction in PXRD peak intensity was observed, which may be attributed to minor crystallinity loss (Fig. 6). These observations support the structural integrity and chemical stability of the material. The consistent PXRD patterns indicate that the crystalline framework remains intact, while the unchanged FTIR spectra confirm that the framework functional groups associated with carbamate retention were preserved. These findings support the durability and suitability of COF-RC for repeated carbamate extraction and further indicate a relationship between structural integrity and sorbent reusability. The structural stability indicated by the PXRD and FTIR results further supports the suitability of COF-RC as a reusable sorbent for sample preparation across different matrices.

**Fig. 6:**
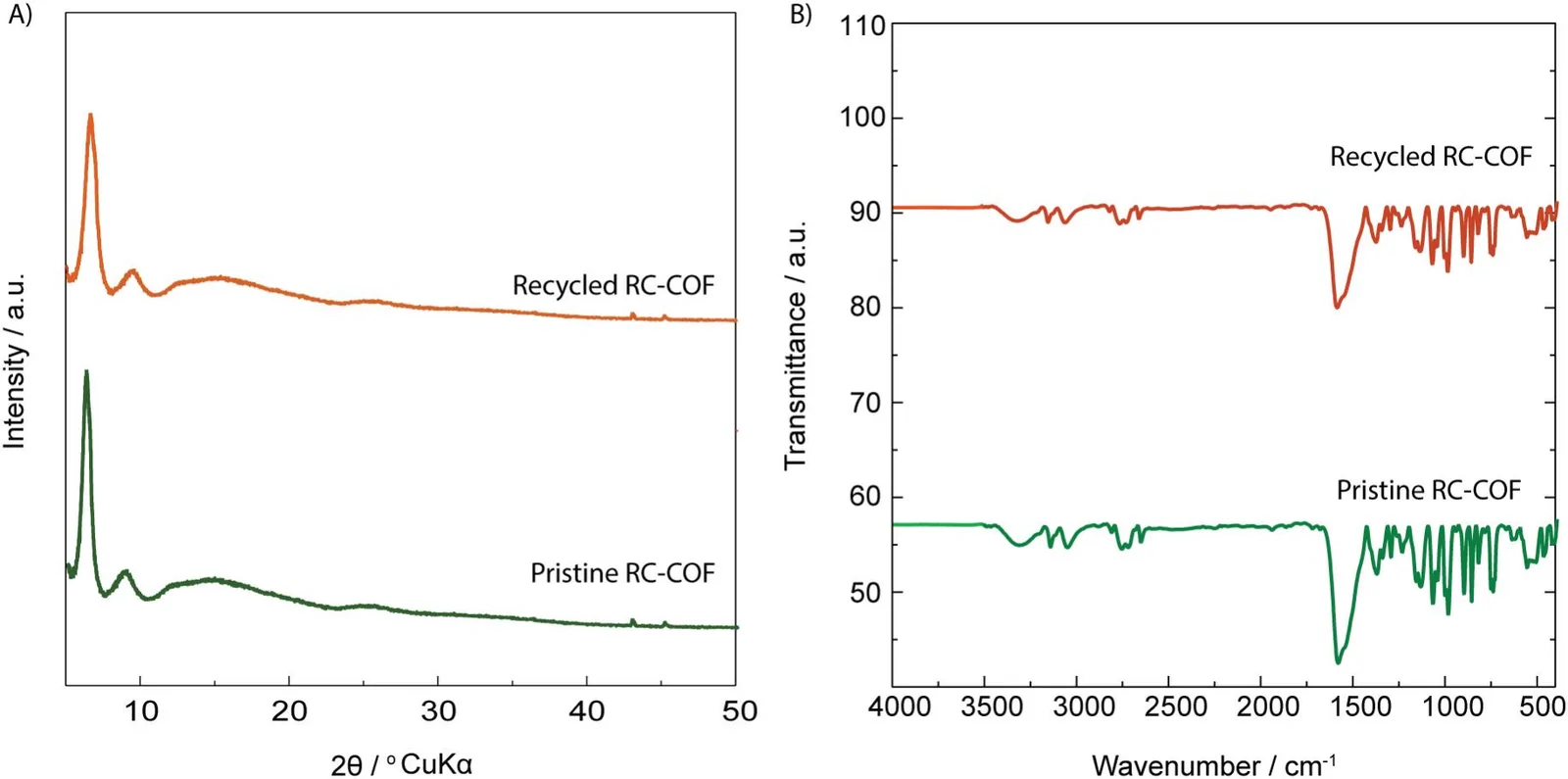
Structural and chemical stability of COF-RC after 10 cycles. (A) PXRD patterns of pristine and recycled RC-COF, demonstrating the retention of crystallinity after multiple adsorption-desorption cycles. (B) FTIR spectra of pristine and recycled RC-COF, confirming that the functional groups responsible for carbamate adsorption remain unchanged, indicating high structural and chemical stability.

The high extraction efficiency and reusability of COF-RC indicate its potential as a cost-effective and reusable SPE sorbent for carbamate residues in food samples. These findings support the applicability of COF-RC as a reliable and reusable sorbent for routine carbamate analysis in food safety laboratories.

### 3.6 Method validation

The performance of the SPE-UHPLC-MS/MS method was validated by evaluating linearity, LOD, LOQ, precision, and matrix effects. As shown in Table S5, excellent linearity was obtained for the carbamates in each fruit matrix, including dates, tomatoes, grapes, and apples, over the validated concentration range. The calibration curves showed high coefficients of determination (R^2^), ranging from 0.9986 to 0.9997 for dates, 0.9990 to 0.9997 for tomatoes, 0.9987 to 0.9994 for grapes, and 0.9985 to 0.9996 for apples, ensuring reliable quantification across the different matrices. The LOD and LOQ values were calculated using the 3σ/k and 10σ/k criteria, respectively. Comparable LOD and LOQ values were obtained across all matrices, ranging from 0.0101 to 0.051 ng/g and 0.0305 to 0.1545 ng/g, respectively, indicating the method's high sensitivity (Table S5).

The precision of the method was assessed through repeatability (intra-day precision) and intermediate precision (inter-day precision, evaluated over three days) by spiking fruit samples with a mixture of carbamate standards at a concentration of 10 ng/g. Precision was expressed as relative standard deviation (RSD). As shown in Table S6, repeatability RSD values ranged from 1.55 to 7.82%, while intermediate precision RSD values ranged from 0.38 to 9.42%, indicating good consistency and reproducibility over multiple days.

Matrix effects for the seven carbamate pesticides in the different fruit matrices were evaluated by comparing the slopes of matrix-matched and solvent-based calibration curves using the following equation:

$$\text{Matrix effect}=\frac{\text{slope}\;\left(\text{matrix}-\text{matched calibration curve}\right)-\text{slope}\;\left(\text{solvent}-\text{based calibration curve}\right)}{\text{slope}\;\left(\text{solvent}-\text{based calibration curve}\right)}\;x\;100$$

Negative matrix effect values indicate ion suppression, whereas positive values indicate ion enhancement. Matrix effect values ranging from −20% to 20% are considered negligible, whereas values exceeding ±20% indicate moderate or strong matrix effects (Kim et al., 2023).

The calculated matrix effects varied among the carbamates and fruit matrices (Table S5). The majority of analyte-matrix combinations exhibited negligible matrix effects within ±20%. Moderate ion enhancement was observed for pirimicarb in dates (43.97%) and tomatoes (37.32%) and for fenoxycarb in dates (36.70%). In contrast, fenoxycarb showed moderate ion suppression in apples (−26.39%). No strong matrix effects were observed for any of the investigated carbamates. These results indicate that matrix effects were generally limited; however, their occurrence for some carbamates supports the use of matrix-matched calibration for reliable quantification across the different fruit matrices.

### 3.7 Application to real samples

The applicability of the developed SPE-UHPLC-MS/MS method to the investigated fruit matrices was further demonstrated through recovery experiments using spiked dates, tomatoes, grapes, and apples. Method accuracy was evaluated by analyzing spiked fruit samples at three concentration levels (0.5, 10, and 100 ng/g), each in five replicates (*n* = 5) (Table 1). The obtained recoveries and corresponding RSD values were 92.7–101.5% with RSDs of 1.6–7.6% for dates, 93.7–100.8% with RSDs of 1.7–11.6% for tomatoes, 95.1–101.5% with RSDs of 2.2–8.2% for grapes, and 94.1–102.9% with RSDs of 1.6–6.8% for apples. All recovery and precision values complied with the acceptable limits established by the European SANTE/11312/2021 guidelines (European Commission, 2021), confirming the reliability and applicability of the method for carbamate residue analysis in fruit matrices.

**Table 1:** Recoveries of the carbamates from fruit samples spiked at different concentration levels (*n* = 5).

Sample	Spiked ng/g	Recovery (%) ± RSD (%)						
		Carbosulfan	Propoxur	Pirimicarb	Bendiocarb	Fenoxycarb	Carbofuran	Methiocarb
Date	0.5	100.6 ± 5.0	98.2 ± 4.6	101.5 ± 6.7	97.6 ± 5.6	99.5 ± 7.6	99.8 ± 7.5	97.7 ± 4.4
	10	99.0 ± 3.9	99.3 ± 3.8	96.9 ± 5.8	95.0 ± 2.4	96.6 ± 5.1	101.4 ± 1.6	99.7 ± 6.6
	100	100.2 ± 3.1	92.7 ± 7.4	100.6 ± 4.2	93.6 ± 3.3	99.0 ± 6.5	97.6 ± 3.2	96.7 ± 2.6

Tomato	0.5	100.1 ± 1.7	99.6 ± 3.7	98.4 ± 3.2	93.7 ± 12	96.5 ± 6.8	99.5 ± 4.3	97.8 ± 2.7
	10	99.1 ± 2.4	97.5 ± 2.6	95.3 ± 3.9	100.0 ± 7.8	100.2 ± 3.6	99.3 ± 4.2	98.0 ± 6.2
	100	95.8 ± 2.2	98.3 ± 5.4	100.8 ± 5.4	100.5 ± 3.5	99.7 ± 6.9	97.5 ± 3.5	99.8 ± 6.5

Grape	0.5	99.0 ± 5.5	100.6 ± 4.9	93.6 ± 3.0	100.7 ± 2.6	99.4 ± 4.0	100.1 ± 3.8	95.8 ± 8.2
	10	100.4 ± 4.0	98.1 ± 6.0	95.3 ± 5.4	100.0 ± 4.0	97.7 ± 4.0	100.7 ± 5.9	100.1 ± 6.7
	100	98.2 ± 7.3	97.0 ± 4.8	98.4 ± 4.4	101.5 ± 3.3	97.5 ± 2.7	100.0 ± 3.4	95.1 ± 8.1

Apple	0.5	99.5 ± 4.0	97.1 ± 4.3	99.0 ± 2.3	97.2 ± 4.3	99.4 ± 3.9	94.1 ± 4.9	98.1 ± 4.6
	10	102.9 ± 6.5	94.2 ± 6.0	97.5 ± 6.6	99.4 ± 6.3	101.0 ± 5.8	96.1 ± 2.3	99.3 ± 1.6
	100	99.5 ± 4.3	98.2 ± 6.8	97.8 ± 5.2	96.7 ± 6.6	96.1 ± 4.9	97.9 ± 2.6	102.3 ± 4.8

Following the satisfactory recovery results, the method was applied to the analysis of unspiked fruit samples to evaluate its practical applicability for carbamate residue determination. The detected concentrations of carbamate residues are presented in Table S7. Representative MRM chromatograms obtained from Sample 2 of each investigated fruit matrix are presented in Fig. S14. Among the analyzed samples, fenoxycarb (3.27–10.02 ng/g) and propoxur (0.76–6.02 ng/g) were the most frequently detected residues, suggesting their prevalent use or persistence in fruit matrices. Other carbamate residues were detected at varying frequencies and concentrations. Carbosulfan was found in one date sample (1.68 ± 0.10 ng/g) and two tomato samples (0.53 ± 0.08 ng/g, 0.99 ± 0.07 ng/g). Pirimicarb was detected in dates, apples, and grapes, with concentrations ranging from 0.34 to 0.40 ng/g. Bendiocarb and methiocarb were only present in grapes and tomatoes, with concentrations of 0.35–0.47 ng/g and 0.17–0.26 ng/g, respectively. Carbofuran was detected in dates, apples, and tomatoes at concentrations ranging from 0.12 to 3.32 ng/g. Overall, the satisfactory recovery performance across the four fruit matrices, together with the successful determination of carbamate residues in unspiked samples, demonstrates the practical applicability of the developed SPE-UHPLC-MS/MS method for trace-level carbamate analysis in complex food matrices.

### 3.8 Comparison with previously reported methods

The proposed COF-RC-based SPE approach coupled with UHPLC-MS/MS demonstrated excellent analytical performance for carbamate analysis compared with other reported methods. Our method achieved remarkably low LODs (0.01–0.051 ng/g), comparable to or lower than those reported for most of the compared techniques, highlighting its high sensitivity (Table 2). For instance, the obtained LODs were substantially lower than those reported for CTAB-modified zeolite NaY-μ-SPE (Salisaeng et al., 2016). The method also required a relatively low sorbent dosage (10 mg) and moderate organic-solvent consumption (2.5 mL methanol), further supporting its practical applicability. Furthermore, the developed method enables the simultaneous determination of seven carbamate pesticides, providing broader multiresidue coverage than most of the previously reported methods summarized in Table 2, many of which were limited to fewer target compounds. In addition to this broader analytical scope, COF-RC-SPE achieved high and relatively narrow recoveries of 92.7–102.9%. This recovery range compares favorably with those reported for several previous methods, including MCOF-DhaTab-MSPE (73.8–88.9%), Fe-Al MMH@MOF-MSPE (71.5–122.8%), and COF-TpDB-SPE (80.4–101.2%). Although the recently reported COF-COOH@MIPs-SPE method showed a narrower recovery range of 100.64–102.78% (Tian et al., 2025), that method was evaluated in water samples, whereas the present method maintained high recoveries across four complex fruit matrices containing substantially greater matrix interferences.

**Table 2:** Comparison of COF-RC with reported sorbents for carbamate pesticide analysis.

Analytical Technique (Extraction-Detection)	Sorbent	Carbamates Studied	Sorbent amount (mg)	Extraction time	Elution solvent/volume	Elution time	LOD (ng/g)	Recovery (%)	Reference
**SPE-UHPLC-MS/MS**	**COF-RC as sorbent for SPE**	**Carbosulfan, propoxur, pirimicarb, bendiocarb, fenoxycarb, carbofuran,and methiocarb**	10	30 min	MeOH, 2.5 mL	15 min	**0.010–0.051**	**92.7–102.9**	**This Study**
MSPE-HPLC-UV	MCOF-DhaTab as magnetic sorbent for MSPE	Carbaryl, fenobucarb, and isoprocarb,	10	5 min	MeOH, 0.6 mL	5 min	0.1–200	73.8–88.9	(Tian et al., 2024)
SPE-HPLC-UV	COF-TpDB as sorbent for SPE	Pirimicarb, carbaryl, methiocarb, and diethofencarb	25	20 min†	ACN, 1.5 mL	5 min	0.005–0.05	80.4–101.2	(Guo et al., 2022)
MSPE-HPLC-UV–Vis	Fe-Al MMH@MOF as magnetic sorbent for MSPE	Bendiocarb, carbosulfan, carbofuran, carbaryl, propoxur, isoprocarb, and promecarb	10	45 s	ACN, 3 mL	45 s	0.005–0.090	71.5–122.8	(Gamonchuang et al., 2022)
SPE-HPLC-DAD	Porous organic polymer as sorbent for SPE	Metolcarb, diethofencarb, isoprocarb, bassa, and carbaryl	20	20 min†	ACN, 0.3 mL	1.6 min†	0.06–0.40	82–110	(Wang et al., 2019)
MSPE-HPLC-UV	Polymeric ionic liquid as magnetic sorbent (Fe_3_O_4_@SiO₂@(PSS-PIL)_10_) for MSPE	Chlorpropham	160	30 s	ACN, 1 mL	2 min	0.5	58.9	(He et al., 2017)
VA-D-μ-SPE-HPLC-DAD	CTAB-modified zeolite NaY as sorbent for μ-SPE	Oxamyl, methomyl, aldicarb, propoxur, bendiocarb, carbaryl, isoprocarb, methiocarb, and promecarb	40	2 min	MeOH, 0.5 mL	NR	4–4000	79.5–124.0	(Salisaeng et al., 2016)
μ-SPE-HPLC-UV–vis	Graphene-modified TiO_2_ nanotube arrays as sorbent for μ-SPE	Metolcarb, carbaryl, isoprocarb, and diethofencarb	Array sheet/ mass NR^1^	60 min	MeOH, volume NR	8 min	2.27–3.26	83.9–108.8	(Zhou & Fang, 2015)
SPE-HPLC-UV	COF-COOH@MIPs as sorbent for SPE	Propoxur, carbofuran, pirimicarb, carbaryl, and methomyl	NR	NR	MeOH:H₂O (60:40, *v*/v), 2 mL	NR	39–186	100.64–102.78	(Tian et al., 2025)
MSPE-Fluorescence	COF (TpPa-NH₂)@Fe_3_O_4_	carbaryl	4	5 min	ACN/B-R buffer (1:1, v/v), 0.4 mL	6 min	0.012	96.0–107.4	(Du et al., 2022)

1 NR: Not reported.

† Calculated from the reported sample volume and loading flow rate.

## 4 Conclusion

In summary, COF-RC was successfully synthesized through a Schiff-base condensation reaction between L1 and L2, yielding a well-ordered 2D crystalline framework with high crystallinity, microporosity, and good chemical and thermal stability. The material showed preferential retention of carbamate pesticides, driven primarily by hydrogen bonding, π–π stacking, and electrostatic interactions. When employed as a sorbent in solid-phase extraction, COF-RC showed good analytical performance, achieving recoveries of 92.7–102.9% across four fruit matrices (dates, tomatoes, grapes, and apples). The framework also showed good structural durability, retaining its extraction efficiency over at least 10 consecutive reuse cycles without significant loss in performance. Coupled with UHPLC-MS/MS detection, the developed COF-RC-SPE method showed good sensitivity, reproducibility, and applicability across the investigated matrices, supporting its potential as a practical tool for routine carbamate residue monitoring in food safety applications. These findings support the potential of COF-RC as a reusable sorbent for pesticide residue analysis and suggest broader applicability in environmental and agri-food monitoring.

## Declaration of generative AI and AI-assisted technologies in the manuscript preparation process

During the preparation of this work the author used ChatGPT in order to enhance language. After using this tool/service, the authors reviewed and edited the content as needed and take full responsibility for the content of the published article.

## CRediT authorship contribution statement

**Rana Morsi:** Writing – original draft, Visualization, Validation, Methodology, Investigation, Formal analysis, Data curation. **Shaikha S. AlNeyadi:** Writing – review & editing, Supervision, Resources, Methodology, Data curation, Conceptualization. **Bayan Al-Meetani:** Validation, Investigation. **Osama Shekhah:** Writing – review & editing, Methodology, Data curation. **Mohammed A. Meetani:** Writing – review & editing, Supervision, Resources, Project administration, Funding acquisition, Conceptualization.

## Funding

This work was supported by the Research Office of the 10.13039/501100006013United Arab Emirates University [fund # 31S462] [fund # 12S290], and [fund # 12R389].

## Declaration of competing interest

The authors declare that they have no known competing financial interests or personal relationships that could have appeared to influence the work reported in this paper.

## Data Availability

Data will be made available on request.
