# Supplementary material for: Facile synthesis of a covalent organic framework for efficient solid-phase extraction and analysis of carbamate pesticide residues in fruits
Source: Food Chem X. 2026 Oct 24;40:104666. doi: 10.1016/j.fochx.2026.104666 (PMC13737719; doi:10.1016/j.fochx.2026.104666)
Supplement: Supplementary file 1 [file mmc1.docx]

**Facile Synthesis of a Covalent Organic Framework for Efficient Solid-Phase Extraction and Analysis of Carbamate Pesticide Residues in Fruits**

Rana Morsi, Shaikha S. AlNeyadi^*^, Bayan Al-Meetani, Osama Shekhah, and Mohammed A. Meetani^*^

R. Morsi, S.S. AlNeyadi, M.A. Meetani: Chemistry Department, College of Science, United Arab Emirates University, Al-Ain, P.O. Box 15551, United Arab Emirates

B. Al-Meetani: Chemical Engineering Department, College of Engineering, United Arab Emirates University, Al-Ain, P.O. Box 15551, United Arab Emirates

O. Shekhah: Advanced Membranes and Porous Materials Center Physical Science and Engineering Division, King Abdullah University of Science and Technology, Thuwal, 23955-6900, Saudi Arabia

#### **Corresponding Authors:**

**Shaikha S. AlNeyadi** (Email: **shaikha.alneyadi@uaeu.ac.ae**; Phone: **+971 3 7136132**)

**Mohammed A. Meetani** (Email: **mmeetani@uaeu.ac.ae**; Phone: **+971 3 713 6136**)

**Fig. S1.** Synthesis of **L1**.

**Fig. S2.** ^1^H-NMR spectrum of L1.

**Fig S2**. ^13^C-NMR spectrum of L2

**Fig. S3.** ^13^C-NMR spectrum of L1.

**Fig. S4.** Synthesis of **L2.**

**Fig. S5.** ^1^H-NMR spectrum of L2.

**Fig. S6.** ^13^C-NMR spectrum of L2.

**Fig. S7.** Experimental PXRD pattern of COF-RC (red) compared with simulated diffraction profiles for AB-stacked (blue) and AA-stacked (black) structural models. The experimental data show a dominant reflection at low angle (≈6–8°) consistent with the (100) plane, along with weaker higher-order reflections. The AB stacking model exhibits much better agreement with the experimental pattern, whereas the AA model predicts sharper peaks not observed experimentally, indicating that COF-RC preferentially adopts an AB-staggered stacking arrangement.

**Fig. S8.** Structural modeling and simulated PXRD patterns of COF-RC. (Left) Unit cell and pore channel representation highlighting a pore size of ~12.5 Å, together with corresponding simulated PXRD pattern. (Right) Structural models showing possible AA (eclipsed) and AB (staggered) stacking arrangements and their respective simulated PXRD patterns. The AB-stacking simulation provides better agreement with the experimental PXRD, reproducing the main low-angle (100) reflection and higher-order peaks, while the AA-stacking model predicts sharper peaks not observed experimentally.

A)

B)

**Fig. S9.** Stability of COF-RC: (A) PXRD stability test showing structural robustness in various solvents and extreme pH; and (B) TGA revealing thermal stability up to ~500°C.

**Fig. S10.** Proposed adsorption mechanism of propoxur on β-ketoenamine-linked COF-RC. The hydroxyl groups and nitrogen-containing sites within the β-ketoenamine/triazine framework participate in hydrogen-bonding interactions with the carbonyl, ether, and amino functionalities of propoxur, while π–π stacking between the aromatic rings of propoxur and the conjugated COF framework further enhances adsorption. The cooperative contribution of hydrogen bonding, π–π interactions, and electrostatic interactions is responsible for the high adsorption affinity of COF-RC.

**Fig. S11.** FTIR spectra of RC-COF (green) and RC-COF@Propoxur (red), highlighting the characteristic stretching vibrations of hydroxyl (-OH) and imine (C=N) groups. The observed shifts in the -OH peak from 3458 cm⁻¹ to 3490 cm⁻¹ and in the C=N peak from 1634 cm⁻¹ to 1644 cm⁻¹ upon propoxur adsorption indicate strong hydrogen bonding and electrostatic interactions between RC-COF and propoxur.

A)

B)

**Fig. S12.** XPS survey spectra of (A) pristine COF-RC and (B) COF-RC@Propoxur, showing their surface elemental compositions before and after propoxur adsorption.

**Fig. S13.** Effect of different elution solvents with the addition of FA.

Recovery (%)

| Solvent Type | Carbosulfan | Propoxur | Pirimicarb | Bendiocarb | Fenoxycarb | Carbofuran | Methiocarb |
| --- | --- | --- | --- | --- | --- | --- | --- |
| Methanol + 0.1% FA | 6.86006 | 90.1147 | 98.6644 | 101.341 | 86.9302 | 91.0261 | 92.5442 |
| Methanol-water (3:1) + 0.1% FA | 0.139579 | 102.801 | 93.1678 | 96.1049 | 70.1652 | 91.8014 | 60.5267 |
| Acetonitrile + 0.1% FA | 12.375 | 92.1324 | 102.463 | 101.314 | 83.5438 | 89.2589 | 58.1062 |
| Acetonitrile-water (3:1) + 0.1% FA | 19.9316 | 94.5887 | 98.3963 | 93.2974 | 80.1799 | 88.4103 | 76.1587 |
| Acetonitrile-methanol (1:1) + 0.1% FA | 15.2808 | 90.0407 | 107.094 | 105.585 | 84.8995 | 95.4044 | 62.6441 |

**Fig. S14.** Representative MRM chromatograms of seven carbamate pesticides obtained from unspiked real fruit samples (Sample 2) of dates, apples, grapes, and tomatoes.

Table S1: MS/MS parameters for the seven carbamates and the internal standard.

| **Analyte** | **Precursor ion (M+H)^+^** | **Product ion 1 (m/z)** | **Collision energy (eV)** | **Product ion 2 (m/z)** | **Collision energy (eV)** | **Product ion 3 (m/z)** | **Collision energy (eV)** |
| --- | --- | --- | --- | --- | --- | --- | --- |
| **Carbosulfan** | 381.1 | 117.95 | 21 | 160.10 | 15 | 75.95 | 36 |
| **Propoxur** | 210.15 | 111.00 | 14 | 168.00 | 8 | 92.95 | 26 |
| **Pirimicarb** | 239.10 | 71.90 | 25 | 182.10 | 15 | 85.05 | 27 |
| **Bendiocarb** | 224.15 | 167.00 | 8 | 109.00 | 18 | 81.00 | 35 |
| **Fenoxycarb** | 302.15 | 88.00 | 22 | 116.05 | 12 | 43.95 | 39 |
| **Carbofuran** | 221.95 | 165.00 | 12 | 123.00 | 21 | 55.00 | 28 |
| **Methiocarb** | 226.10 | 169.00 | 9 | 121.00 | 18 | 122.05 | 24 |
| **Pirimicarb d6** | 245.15 | 78.05 | 25 | 185.10 | 15 | 88.05 | 29 |

Table S2: Comparison of the principal XPS binding energies of COF-RC before and after propoxur adsorption.

| **XPS region** | **COF-RC (eV)** | **COF-RC@Propoxur (eV)** | **ΔBE (eV)** |
| --- | --- | --- | --- |
| **C 1s** | ~284.5 | ~284.5 | ~0.0 |
| **N 1s** | ~399.6 | ~399.6 | ~0.0 |
| **O 1s** | ~532.8 | ~532.4 | ~−0.4 |

Table S3: Selectivity evaluation of COF-RC towards carbamates and OPPs.

| Carbamates | | |
| --- | --- | --- |
|  | Recovery (%) | STD |
| Carbosulfan | 100.25 | 1.82 |
| Propoxur | 94.70 | 0.67 |
| Pirimicarb | 95.06 | 1.96 |
| Bendiocarb | 96.87 | 4.97 |
| Fenoxycarb | 101.62 | 1.54 |
| Carbofuran | 101.80 | 3.60 |
| Methiocarb | 99.36 | 2.40 |
| OPPs | | |
| Chlorpyrifos | 7.92 | 1.67 |
| Diazinon | 55.42 | 0.57 |
| Dicrotophos | 5.20 | 0.10 |
| Malathion | 52.78 | 1.17 |
| Parathion | 3.93 | 0.08 |
| Phorate | 1.72 | 0.44 |
| Temephos | 67.67 | 1.81 |

Table S4: Recyclability Performance of COF-RC Over 15 Extraction Cycles.

|  | Cycle 1 | Cycle 2 | Cycle 3 | Cycle 4 | Cycle 5 | Cycle 6 | Cycle 7 | Cycle 8 | Cycle 9 | Cycle 10 | Cycle 11 | Cycle 12 | Cycle 13 | Cycle 14 | Cycle 15 |
| --- | --- | --- | --- | --- | --- | --- | --- | --- | --- | --- | --- | --- | --- | --- | --- |
| Carbosulfan | 95 | 102 | 99 | 102 | 103 | 97 | 98 | 102 | 102 | 99 | 100 | 100 | 98 | 98 | 93 |
| Propoxur | 102 | 97 | 96 | 99 | 99 | 96 | 98 | 97 | 91 | 90 | 90 | 87 | 76 | 76 | 58 |
| Pirimicarb | 103 | 101 | 99 | 103 | 101 | 100 | 97 | 101 | 99 | 92 | 85 | 86 | 78 | 79 | 72 |
| Bendiocarb | 98 | 100 | 100 | 97 | 100 | 101 | 101 | 96 | 100 | 94 | 87 | 81 | 76 | 77 | 67 |
| Fenoxycarb | 96 | 101 | 102 | 97 | 97 | 95 | 97 | 100 | 96 | 102 | 100 | 93 | 86 | 81 | 81 |
| Carbofuran | 101 | 99 | 98 | 98 | 98 | 98 | 103 | 103 | 100 | 99 | 94 | 90 | 89 | 88 | 77 |
| Methiocarb | 100 | 99 | 101 | 96 | 98 | 102 | 102 | 102 | 98 | 102 | 99 | 91 | 79 | 75 | 71 |

Table S5: Validation parameters of the proposed method.

| Carbamate | Matrix | R^2^ | LOD  (ng/g) | LOQ  (ng/g) | Matric effect  (%) |
| --- | --- | --- | --- | --- | --- |
| Carbosulfan | Date | 0.9990 | 0.0132 | 0.0400 | -7.63 |
|  | Tomato | 0.9991 | 0.0127 | 0.0386 | -13.77 |
|  | Grape | 0.9994 | 0.0112 | 0.0339 | 7.36 |
|  | Apple | 0.9996 | 0.0127 | 0.0385 | -12.46 |
| Propoxur | Date | 0.9996 | 0.0139 | 0.0422 | 12.54 |
|  | Tomato | 0.9991 | 0.0147 | 0.0445 | 2.76 |
|  | Grape | 0.9991 | 0.0145 | 0.0439 | 13.24 |
|  | Apple | 0.9990 | 0.0134 | 0.0407 | -16.19 |
| Pirimicarb | Date | 0.9996 | 0.0122 | 0.0371 | 43.97 |
|  | Tomato | 0.9990 | 0.0139 | 0.0422 | 37.32 |
|  | Grape | 0.9994 | 0.0114 | 0.0346 | 5.65 |
|  | Apple | 0.9992 | 0.0115 | 0.0349 | -7.77 |
| Bendiocarb | Date | 0.9990 | 0.0470 | 0.1424 | 4.18 |
|  | Tomato | 0.9990 | 0.0500 | 0.1514 | 17.23 |
|  | Grape | 0.9992 | 0.0470 | 0.1425 | -9.77 |
|  | Apple | 0.9985 | 0.0510 | 0.1545 | 11.52 |
| Fenoxycarb | Date | 0.9991 | 0.0127 | 0.0384 | 36.70 |
|  | Tomato | 0.9991 | 0.0101 | 0.0305 | 20.03 |
|  | Grape | 0.9990 | 0.0149 | 0.0451 | -6.06 |
|  | Apple | 0.9995 | 0.0128 | 0.0389 | -26.39 |
| Carbofuran | Date | 0.9997 | 0.0172 | 0.0520 | 14.81 |
|  | Tomato | 0.9992 | 0.0120 | 0.0362 | 7.28 |
|  | Grape | 0.9993 | 0.0129 | 0.0392 | -8.42 |
|  | Apple | 0.9993 | 0.0102 | 0.0309 | 0.33 |
| Methiocarb | Date | 0.9986 | 0.0148 | 0.0449 | 3.51 |
|  | Tomato | 0.9997 | 0.0139 | 0.0420 | 10.60 |
|  | Grape | 0.9987 | 0.0146 | 0.0442 | 19.81 |
|  | Apple | 0.9993 | 0.0138 | 0.0417 | -7.63 |

Table S6: Intra-day and inter-day precision (RSD%) of carbamate pesticides in different fruit matrices.

| Carbamate | Matrix | RSD % Intraday | RSD%  Interday (n=3) |
| --- | --- | --- | --- |
| Carbosulfan | Date | 3.9 | 2.8 |
|  | Tomato | 2.4 | 2.2 |
|  | Grape | 4.0 | 3.7 |
|  | Apple | 6.5 | 4.7 |
| Propoxur | Date | 3.8 | 1.6 |
|  | Tomato | 2.6 | 2.1 |
|  | Grape | 6.0 | 0.4 |
|  | Apple | 6.0 | 1.4 |
| Pirimicarb | Date | 5.8 | 3.3 |
|  | Tomato | 3.9 | 3.7 |
|  | Grape | 2.7 | 3.2 |
|  | Apple | 6.6 | 3.1 |
| Bendiocarb | Date | 2.4 | 1.5 |
|  | Tomato | 7.8 | 2.3 |
|  | Grape | 4.0 | 2.1 |
|  | Apple | 6.3 | 3.9 |
| Fenoxycarb | Date | 5.1 | 6.1 |
|  | Tomato | 3.6 | 1.6 |
|  | Grape | 4.0 | 1.9 |
|  | Apple | 5.8 | 4.5 |
| Carbofuran | Date | 1.6 | 0.9 |
|  | Tomato | 4.2 | 4.6 |
|  | Grape | 5.9 | 2.8 |
|  | Apple | 2.3 | 1.1 |
| Methiocarb | Date | 6.6 | 3.2 |
|  | Tomato | 6.2 | 2.1 |
|  | Grape | 6.7 | 9.4 |
|  | Apple | 1.6 | 0.9 |

Table S7: Detected concentration of carbamate residues in various fruits employing the proposed SPE-HPLC-MS/MS method.

|  | Concentration ± STD^1^  (ng/g) | | | | | | | | | | | |
| --- | --- | --- | --- | --- | --- | --- | --- | --- | --- | --- | --- | --- |
| Carbamate | Dates | | | Apples | | | Grapes | | | Tomatoes | | |
|  | Sample 1 | Sample 2 | Sample 3 | Sample 1 | Sample 2 | Sample 3 | Sample 1 | Sample 2 | Sample 3 | Sample 1 | Sample 2 | Sample 3 |
| Carbosulfan | ND^2^ | 1.68 ± 0.10 | ND | ND | ND | ND | ND | ND | ND | 0.53 ± 0.08 | 0.99 ± 0.07 | ND |
| Propoxur | 6.02 ± 0.55 | 5.31 ± 0.58 | 4.49 ± 0.88 | 0.76 ± 0.17 | 5.02 ± 0.32 | 4.17 ± 0.51 | ND | 4.52 ± 0.14 | ND | ND | 5.72 ± 0.50 | 3.86 ± 0.29 |
| Pirimicarb | ND | 0.40 ± 0.02 | 0.39 ± 0.05 | ND | ND | 0.36 ± 0.01 | 0.34 ± 0.01 | 0.34 ± 0.01 | ND | ND | ND | ND |
| Bendiocarb | ND | ND | ND | ND | ND | ND | 0.35 ± 0.03 | ND | ND | 0.47 ± 0.11 | ND | ND |
| Fenoxycarb | 3.91 ± 0.67 | 6.61 ± 0.82 | 3.27 ± 0.48 | 5.08 ± 0.04 | 3.82 ± 0.14 | 5.24 ± 0.74 | 3.52 ± 0.21 | ND | 4.16 ± 0.35 | 8.62 ± 0.06 | 10.02 ± 0.05 | 8.13 ± 0.20 |
| Carbofuran | 3.32 ± 0.50 | 0.36 ± 0.02 | ND | ND | 0.12 ± 0.01 | ND | ND | ND | ND | 1.44 ± 0.22 | ND | ND |
| Methiocarb | ND | ND | ND | ND | ND | ND | 0.17 ± 0.05 | ND | 0.21 ± 0.01 | ND | 0.26 ± 0.02 | ND |

^1^STD: standard deviation

^2^ND: not detected
